# From global political agreement to public health practice: operationalising the Belém adaptation indicators for health

**DOI:** 10.1088/1748-9326/ae6674

**Published:** 2026-05-14

**Authors:** Yasna Palmeiro-Silva, Kristie L Ebi, Carole M Green, Haemin Park, Anderson Flomo Jr, Jeremy J Hess

**Affiliations:** Center for Health and the Global Environment, University of Washington, Seattle, WA, United States of America

**Keywords:** climate change, health, indicators, adaptation, climate

## 1. Introduction

Systematic tracking of population health and programming indicators is central to public health science and practice and essential to protecting and improving population health. From London’s Bills of Mortality in the seventeenth century to contemporary global disease surveillance networks, indicators have transformed our capacity to understand, prevent, and respond to population health risks.

However, despite clear evidence of environmental considerations in public health concerns, most public health indicators focus on infectious and non-communicable diseases. Environmental health indicators have been relatively narrowly construed and sometimes seen as secondary and isolated from core public health areas, predominantly being related to exposure to specific chemicals and pesticides, radiation, heavy metals, and air, water, and soil pollution [1].

The growing evidence of population health impacts from exposures to extreme weather, climate variability, and anthropogenic climate change has led to ‘new’ indicators of health hazards and related exposures, such as meteorological and climatic factors [2, 3]. For example, non-optimal temperature represented 9.4% of global deaths, being the 11th leading risk factor in the Global Burden of Disease (GBD) Study when it was introduced in 2019 [4, 5]. Globally, the public health community has pursued several efforts to develop and track climate and health indicators (appendix panel A1), including ones focused on adaptation, but standardised approaches have been lacking. The lack of a standardised framework for climate-health indicators impedes public health practice, especially in low- and middle-income countries (LMIC), where the impacts are most stark.

At the 30th Conference of the Parties (COP30) to the United Nations Framework Convention on Climate Change (UNFCCC), participants adopted the Belém adaptation indicators (BAI), including a slate of health indicators, offering an opportunity to advance climate and health surveillance and programming. The Belém–Addis vision is a subsequent two year political and technical process to refine the indicators for operationalisation and implementation. In this article, we outline definitional, methodological, and practical considerations in operationalising the BAI, offering science-based suggestions for future developments.

## 2. Global goal on adaptation (GGA) and BAI

The Glasgow–Sharm el-Sheikh (GlaSS) work programme (COP26) was tasked with outlining an implementation plan of the GGA of the Paris Agreement [6] The GlaSS ended with the adoption of the UAE Framework for Global Climate Resilience in 2023, pivotal for the development of climate and health indicators. The UAE Framework specified health indicator goals in Target 9(c): ‘Attaining resilience against climate change related health impacts, promoting climate-resilient health services, and significantly reducing climate-related morbidity and mortality, particularly in the most vulnerable communities’ [7].

This was a notable achievement: securing agreement among nearly 200 Parties on the importance of health within the adaptation framework represented a significant political accomplishment and created unprecedented opportunities for the health sector.

The UAE-Belém Work Programme (2024–2025) was charged with specifying indicators to meet the health target. It convened 78 technical experts, including several from the health sector, to develop indicators (appendix panel A2). The process began with over 9000 indicators from Party submissions before progressive reduction to the final framework (aiming for 100 indicators) [8]. For Target 9(c)-Health, Parties submitted 747 proposed indicators that were, through review and revision, distilled down to ten proposed indicators for review at COP30.

At COP30, eight indicators were ultimately adopted as part of the BAI (table 1). The health indicators in the BAI differ substantially from the expert proposal, and the differences are likely to have practical implications that merit consideration. Additionally, the decision text adopted at COP30 states that the indicators *‘shall not create new obligations for developing country Parties, benchmarks or evaluation criteria, nor establish global standardised methodologies or data-collection processes’* [9]. This framing reflects the political compromises necessary to achieve consensus but raises concerns about whether indicators lacking standardised definitions and methods can meaningfully track adaptation progress and outcomes.

**Table 1.** Comparison of expert-proposed (column A) and adopted Belém adaptation indicators for health (column B). Comparison of indicators made to the closest possible meaning.

Column A: Indicators proposed by experts	Column B: Indicators adopted
Change in the rate of mortality associated with heat exposure	Indicator 1: Rate of mortality associated with climate impacts compared with counterfactual rates, including as an outcome of adaptation actions or coverage of early warning systems where applicable
No similar indicator	Indicator 2: Rate of morbidity associated with climate impacts compared with counterfactual rates, including as an outcome of adaptation actions where applicable
Change in the incidence of climate-sensitive infectious diseases (dengue, Chikungunya, Zika, tick-borne encephalitis (TBE), West Nile fever (WNF), malaria and diarrheal diseases)	Indicator 3: Level of incidence of climate-sensitive infectious diseases, including as an outcome of adaptation actions where applicable
Change in the annual rate of reported heat-related occupational injuries and deaths	No similar indicator, though occupational injuries and deaths could be included in Indicator 2.
Extent of implementation of Mental Health and Psychosocial Support (MHPSS) preparedness and response for climate change-sensitive events	Indicator 4: Proportion of the population vulnerable to climate change with access to mental health and psychosocial support
Number of destroyed or damaged health facilities and number of disruptions to health services associated with climate-related events	Indicator 5: Extent to which climate health services have remained at full capacity during and following climate-related events relative to pre-event service capacity
Percentage of health facilities built or retrofitted to be climate resilient based on national, regional or global guidance	Indicator 6: Percentage of health facilities that are resilient to climate-related hazards under different warming scenarios, as appropriate for regions and contexts, including as an outcome of adaptation actions where applicable
Universal Health Coverage (UHC): Coverage of essential health services (SDG 3.8.1)	Indicator 7: Coverage of essential health services that are supported by adaptation measures to ensure continuity during and following climate-related events
Level of operationalisation of climate-informed health early warning systems for climate change-related health risks accessible to vulnerable groups	No similar indicator
Level of implementation of climate change and health vulnerability and adaptation assessment	No similar indicator
Proportion of the ministry in charge of health’s workforce that has received training on climate change and health in the last two years	Indicator 8: Proportion of health practitioners who have received capacity-building support pertaining to climate change adaptation and health

Overall, two main patterns emerge from this comparison. First, there is a consistent shift from specificity to generality, including going from particular exposures (e.g. heat exposure) and diseases (e.g. dengue) to broader terms (e.g. climate impacts, climate-sensitive infectious diseases), that accommodate national discretion but might prove difficult for assessments and comparability. Second, some adopted indicators explicitly introduced additional analytic steps that might add complexity that few countries can currently address, including the analysis of counterfactual comparisons and the potential consequences of adaptation actions. This divergence between expert recommendations and political outcomes is consistent with documented patterns in the science–policy interface. Research on UNFCCC negotiations reveals that, even when scientific consensus is strong, policy recommendations routinely diverge, reflecting the need to balance policy considerations suggested by the best available science with considerations of national sovereignty, economic interests, implementation capacity, practicality, and negotiating dynamics [10, 11]. The result of negotiations on the BAI reflects these tensions, at least, for health and will require continued assessment of the practical nature of how these indicators will be measured and used.

## 3. Reflections on the BAI for health

For each adopted BAI for health, we assess five dimensions of operational viability: definitional considerations, assessment approaches, data requirements and sources, counterfactual and adaptation considerations, and implementation considerations (table 2). We selected these dimensions because they collectively span the full operationalisation pathway of any public health indicator, from conceptual clarity and methodological rigour through to practical feasibility and therefore provide a systematic basis for identifying where further technical development is needed. We offer a proposed starting point for tracking indicators of this scale and specificity; and for iterative review. Illustrative operationalisation examples for selected indicators are provided in appendix table A1.

**Table 2.** Indicator-specific reflections and considerations.

**Indicator 1: Rate of mortality associated with climate impacts compared with counterfactual rates, including as an outcome of adaptation actions or coverage of early warning systems where applicable**	
*Definitional considerations*	This indicator requires specification regarding the climate impacts to be included and the counterfactual(s) against which observed mortality is compared. It is not clear whether the indicator conflates ‘climate impacts’ with ‘climate change impacts’, though tracking mortality associated with heat (weather-related) is methodologically distinct from tracking mortality attributable specifically to heat caused by anthropogenic climate change (climate change-attributable).
*Assessment approaches*	Methodological approaches to this indicator include event-based approaches that calculate excess mortality during defined events such as heatwaves or floods, requiring definition criteria (e.g. temperatures exceeding the 95th percentile of a specified baseline period for three consecutive days), expected mortality baselines, and observed mortality during the event period [14]. A second approach is time-series, including the use of generalised additive models or distributed lag non-linear models, to estimate exposure-response relationships between weather and climate variables and health outcomes, capturing acute and lagged effects.
*Data requirements and sources*	Comprehensive health data are required to model these impacts. Data requirements include daily cause-specific mortality counts, age, sex, and geographic unit. Exposure data include daily weather observations (depending on the ‘climate impact’ under analysis) at sufficient spatial resolution, for example reanalysis products. Covariates include population denominators, socioeconomic indicators, and air quality data for confounding adjustment. Data sources include Civil Registration and Vital Statistics systems for mortality (or other health outcomes) and meteorological services for weather or climate exposure. At minimum, weekly or monthly all-cause mortality counts linked to daily temperature could provide initial estimates, though coarser temporal resolution attenuates acute effect detection, and all-cause mortality dilutes the climate-specific signal, increasing uncertainty in effect estimates.
*Counterfactual and adaptation considerations*	Depending on the purpose of this indicator, establishing baseline periods or another plausible scenario is needed for counterfactual comparison. To include adaptation and early warning systems in the analysis, data on the absence or presence of the specified intervention are required; data on intervention effectiveness are required for counterfactual analyses.
*Implementation considerations*	Operationalisation requires epidemiological expertise in environmental health and linkage between health and meteorological data systems. Equity considerations require disaggregation by socioeconomic status, geographic area, and vulnerability factors.

**Indicator 2: Rate of morbidity associated with climate impacts compared with counterfactual rates, including as an outcome of adaptation actions where applicable**	

*Definitional considerations*	This indicator mirrors Indicator 1 but for non-fatal health outcomes. ‘Morbidity’ requires specification as it might include disease incidence, healthcare utilisation (i.e. emergency department visits, hospitalisations, primary care consultations), or disability burden. Morbidity is more difficult to measure consistently than mortality due to greater variability in case definitions, healthcare-seeking behaviour, and data capture systems. However, morbidity indicators capture sub-lethal impacts that mortality misses, making them particularly important for conditions that rarely result in death but cause substantial burden.
*Assessment approaches*	Definitions of morbidity cases are needed. Healthcare utilisation would track, for example, emergency department visits and hospitalisations for health outcomes such as heat stroke, heat exhaustion, dehydration, respiratory disease during pollution events, and injuries during floods. This has the advantage that data are often available from healthcare administrative systems, if there is a registering system in place. Analyses follow the same fashion as indicator 1.
*Data requirements and sources*	Healthcare data include clinic and emergency department records hospital discharge records, and primary care records with diagnosis codes, date, location, and patient demographics. Survey data from population health surveys capturing self-reported illness, healthcare access, and functional limitations might provide complementary information, but temporal coverage and standardisation is limited. Data sources include hospital information systems, insurance claims data where coverage exists, and household surveys such as the Demographic and Health Surveys. At minimum, aggregated monthly hospital discharge data or syndromic surveillance reports could be used, though reduced temporal granularity and broader case definitions increase misclassification and limit detection of acute exposure-response relationships.
*Counterfactual and adaptation considerations*	These considerations follow the same logic as Indicator 1. Key additional considerations include the importance of specifying which health outcomes are included and defining adaptation actions specific to morbidity prevention such as access to cooling during heatwaves or treatment availability for climate-sensitive conditions. Implementation requires investment in health information systems with diagnostic coding and capacity to link healthcare data with weather and climate exposures.
*Implementation considerations*	Same considerations as Indicator 1.

**Indicator 3: Level of incidence of climate-sensitive infectious diseases, including as an outcome of adaptation actions where applicable**	

*Definitional considerations*	The indicator refers only to ‘climate-sensitive infectious diseases’ without specification, leaving a gap in terms of which diseases qualify. This flexibility may accommodate national contexts but undermines comparability across different jurisdictions and over time if case definitions change. A tiered disease list would address this: core diseases for global tracking (e.g. dengue, malaria, cholera/acute watery diarrhoea), regional diseases (e.g. tick-borne encephalitis, West Nile in temperate zones; Chikungunya, Zika in tropical/subtropical regions), and context-specific additions where countries demonstrate local climate sensitivity (accompanied by a clear definition of cases).
*Assessment approaches*	Incidence should be calculated as suspected and confirmed cases per 100 000 population per year and age-standardised for comparability. Climate linkage analyses should correlate incidence trends with climate variables using time-series methods and document whether observed trends are consistent with climate projections such as poleward range expansion of vectors [15]. A key challenge is distinguishing climate-driven changes from other drivers including land use change, urbanisation, control programme effectiveness, and population immunity, requiring additional data and more advanced modelling and analysis.
*Data requirements and sources*	Data requirements include suspected and confirmed cases by disease, date, location, age, and sex (and other variables of interest), with laboratory confirmation preferred but clinical diagnosis acceptable where appropriate. Population denominators, weather and climate data, and vector surveillance data where relevant are also needed. Data sources include national disease surveillance systems and meteorological data systems. As underreporting might vary substantially by disease and setting [16], adjustment of methods might be required for comparability. At minimum, routine notifiable disease reports at weekly or monthly resolution with climate data could support trend analysis, though underreporting, inconsistent case definitions, and diverse temporal aggregation introduce substantial uncertainty in associations.
*Counterfactual and adaptation considerations*	Counterfactual analyses follow similar observations as previous indicators. If the aim is to disentangle the effect of multiple factors, including secular improvements in the health systems and socioeconomic development, options include trend analysis adjusting for non-climate factors, or comparison of observed incidence to model predictions based on climate suitability. Declining incidence might indicate successful adaptation even as climate suitability increases, therefore, analyses of both climate suitability and cases are preferred. Disaggregating adaptation actions is a consideration as incidence of climate-sensitive infectious diseases might be lower in settings where adaptations are prevalent [17]. Adaptation actions include vector control programmes, water, sanitation and hygiene improvements, early warning systems, and vaccination where applicable.
*Implementation considerations*	Most countries have surveillance capacity for some climate-sensitive diseases through International Health Regulations (IHR) requirements, providing a foundation for implementation. The indicator aligns strongly with SDG 3 and IHR. Building on existing disease surveillance infrastructure with added climate data linkage and analysis capacity represents a feasible pathway forward [18].

**Indicator 4: Proportion of the population vulnerable to climate change with access to mental health and psychosocial support**	

*Definitional considerations*	This indicator requires definition of three main concepts. The first relates to vulnerability. Second, ‘access’ to services requires operationalisation and specification: geographic access (e.g. physical proximity, travel time), financial access (e.g. affordability, insurance coverage), or effective access (e.g. actual utilisation, quality of care received, social behaviour). Third, ‘mental health and psychosocial support’ encompasses a spectrum from community-based psychosocial programmes to specialist psychiatric services, as captured in the Inter-Agency Standing Committee (IASC) Mental Health and Psychosocial Support (MHPSS) pyramid [19]. Without standardised definitions, countries will measure different things. Notably, this indicator uses ‘climate change’ rather than ‘climate impacts’, introducing inconsistency with other indicators or the idea of intentionally measuring long-term vulnerability and attribution to anthropogenic climate change.
*Assessment approaches*	The indicator formula would be: (Vulnerable population with MHPSS access/Total vulnerable population) × 100. A practical approach would use service availability mapping to assess MHPSS service presence, combined with population vulnerability mapping based on climate hazard exposure and socioeconomic indicators. It would be ideal to spatially and temporally map these metrics, so decision-makers are able to evaluate trends, progress, and areas that need specific support.
*Data requirements and sources*	Vulnerability mapping requires climate vulnerability indices, socioeconomic data, and population distribution data. Service mapping might require Mental Health Atlas data from the World Health Organisation (WHO), national mental health service registries, and/or Service Availability and Readiness Assessment (e.g. WHO SARA tool) data. Utilisation data requires health facility records and household surveys on mental health service use. A critical gap is that mental health services are severely under-resourced in most low- and middle-income countries. At minimum, national-level estimates of MHPSS service availability combined with population counts in at-risk zones could provide a crude coverage estimate, though lack of subnational granularity and utilisation data limits interpretation of actual access.
*Counterfactual and adaptation considerations*	If the indicator is aiming to compare the proportion of the population vulnerable to climate change, then counterfactual scenarios that include no climate change signal are needed.
*Implementation considerations*	This indicator is inherently complex due to the multiple contested definitions as described above. A tiered approach may be needed: basic level measuring presence of any MHPSS services in climate-vulnerable areas; intermediate level estimating coverage based on service capacity versus population need; and advanced level tracking utilisation and outcome data for vulnerable populations following climate events.
**Indicator 5: Extent to which climate health services have remained at full capacity during and following climate-related events relative to pre-event service capacity**	

*Definitional considerations*	This indicator introduces undefined terminology that needs clarification. ‘Climate health services’ could mean services specifically addressing climate-sensitive or related conditions (e.g. heat illness, vector-borne disease), all health services during climate events, health services with climate-resilience features, weather and climate services that inform health decision-making, or just health services generally, which are responsible for care for climate-sensitive conditions and diseases of other aetiologies. Clarification is essential for consistent measurement. ‘Full capacity’ has multiple dimensions including structural (e.g. physical infrastructure intact and operational), functional (e.g. services being delivered), workforce (e.g., staff availability and deployment), supply chain (e.g. medicines, equipment, power, water available), and quality of care (e.g. a system may continue to provide care at the same level of access but reduce quality standards). In addition, the duration of the event needs to be specified—the weather-related disturbance may last a day, but recovery may take years.
*Assessment approaches*	Depending on how ‘climate health services’ and ‘capacity’ are defined, a specific service capacity index might be needed, for example: (Service output during/after event/Pre-event baseline service output) × 100. Service output could be measured by outpatient consultations, inpatient admissions, surgical procedures, immunisations delivered, or a composite index. An alternative binary assessment would determine whether the facility was operational during and 30 days (or more) following the event. Importantly, the indicator presumes pre-event or baseline data that many health systems lack, requiring investment in routine service delivery monitoring.
*Data requirements and sources*	Service delivery data from health management information systems and facility registers are required. Event exposure data from disaster databases (e.g. EM-DAT, Desinventar) and meteorological records must be linked to facility locations through facility lists with geolocation. A key challenge is that real-time data on service disruption are often not captured systematically, and retrospective assessment may introduce recall bias. Other complicating factors relate to the complexity of health systems and public, private, and mixed systems with different reporting requirements and capabilities, and reluctance of health systems to provide transparent data on operational factors and quality of care. At minimum, post-event facility damage assessment and qualitative service disruption could provide binary indicators of impact, though retrospective collection introduces recall bias and absence of baseline data precludes quantitative continuity indices.
*Counterfactual and adaptation considerations*	This indicator focuses on short-term resilience (i.e. maintaining services during events) and long-term adaptation. Consideration should be given to distinguishing immediate impact (e.g. service disruption during event), recovery trajectory (e.g. time to return to baseline), and build-back-better (e.g. whether capacity improves post-event). An opportunity is to evaluate the effect of adaptation interventions on the service capacity (depending on definitions).
*Implementation considerations*	The indicator aligns with Sendai Framework indicators on health facility damage [20] and should be harmonised with existing local disaster impact assessment approaches.
**Indicator 6: Percentage of health facilities that are resilient to climate-related hazards under different warming scenarios, as appropriate for regions and contexts, including as an outcome of adaptation actions where applicable**	

*Definitional considerations*	A clear definition of ‘health facilities’ is needed (e.g. general hospitals, primary care centres, rural health centres) and whether supply chains are being included. ‘Resilience’ is a complex concept with multiple dimensions: structural resilience (e.g. building withstands physical impacts from wind, flood, heat), functional resilience (e.g. services continue during disruption), and adaptive capacity (e.g. ability to adjust to changing conditions). ‘Different warming scenarios’ implies forward-looking assessment against 2 °C and higher warming scenarios, requiring climate projections, socioeconomic scenarios and vulnerability assessments, and specific modelling scenarios as well as elaboration of health system characteristics across the Shared Socioeconomic Pathways. A critical gap is that no globally accepted standard for ‘climate-resilient health facility’ currently exists. The WHO has an operational framework for climate-resilient health systems [13], but no standardised scheme for quantifying climate-resilient health systems has been established. As with other BAI indicators, if adaptation is to be considered, activities and implementation parameters will need to be specified.
*Assessment approaches*	Options include a checklist approach developing standardised resilience criteria (e.g. structural integrity, water/sanitation, power, supply chain, workforce) and assessing facilities against it; a risk assessment approach mapping facility locations against climate hazard exposure, assessing vulnerability, and rating resilience; or a performance-based approach tracking facility performance during past climate events as a proxy for resilience.
*Data requirements and sources*	A complete facility inventory with structural and operational characteristics is the foundation. Hazard exposure requires climate hazard maps (current and projected), flood zone maps, and heat projections, for example. Resilience assessment requires facility-level resilience checklist data, which would need new data collection. Adaptation assessment requires information regarding implementation of priority interventions within and outside the health sector. Most countries lack complete facility registries with structural information, and the assessment methodology requires development and validation. There are no existing lists of essential adaptations or surveillance activities related to their implementation. At minimum, a master facility list with geolocation combined with self-reported resilience checklists could yield initial estimates, though self-assessment without validation against actual event performance introduces substantial measurement uncertainty.
*Counterfactual and adaptation considerations*	The indicator considers a modelling exercise of resilience (and potentially diverse dimensions of resilience, each affected by different hazards) in terms of warming scenarios. It is not explicit in the indicator, but different adaptation interventions that affect (or modify) resilience can be included in the modelling.
*Implementation considerations*	This is perhaps the most challenging indicator to operationalise due to definitional complexity and multiple data gaps. Climate resilience is not only a facility-specific variable; it is a function of capacities and mutual aid operating at community and regional levels simultaneously, as well as management of supply chain issues that extend globally. A phased approach might be useful: Phase 1 completing facility registries with geolocation; Phase 2 conducting hazard exposure mapping; Phase 3 developing and validating resilience assessment methodology; Phase 4 implementing systematic facility assessment. The indicator aligns with WHO climate-resilient health systems framework and Sendai targets on critical infrastructure.
**Indicator 7: Coverage of essential health services that are supported by adaptation measures to ensure continuity during and following climate-related events**	

*Definitional considerations*	‘Essential health services’ can span a wide range and needs clear definition. ‘Supported by adaptation measures’ requires judgement about which measures qualify as adaptation and what ‘supported’ means: direct financing, policy mandate, or infrastructure investment. ‘Continuity’ during climate-related events also requires operational definitions. The key ambiguity is whether this measures, for example, coverage of essential services that have been designated ‘climate-proofed’, coverage of services during climate events, or the existence of adaptation measures for essential services.
*Assessment approaches*	A policy-focused approach would assess whether essential health services have documented adaptation or continuity plans, yielding binary or a scored assessment. An outcome-focused approach would track a service coverage index (or continuity index) during and after climate-related events versus pre-event or normal periods, measuring actual continuity. Separating into two sub-indicators may be an option: percentage of essential health services with documented adaptation or continuity measures, and maintenance of essential service coverage during climate events compared to a baseline.
*Data requirements and sources*	Assessment of adaptation measures requires policy review and programme documentation. Service continuity tracking can use existing health management information system data if disaggregated by time period relative to climate-related events. At minimum, policy documentation review to assess presence of adaptation or continuity plans for essential services (yielding binary assessments) is feasible, through this captures intent rather than implementation or effectiveness.
*Counterfactual and adaptation considerations*	Potential scenario comparison might include services with limited support and the continuity index. Adaptation considerations include an array of potential interventions, each with specific and compounding effects. Counterfactual frameworks need to account for divergent levels of adaptation investment and implementation as climate-sensitive disease impacts accrue.
*Implementation considerations*	The indicator aligns with SDG 3.8.1 and existing essential health services monitoring frameworks, providing opportunities for harmonisation.

**Indicator 8: Proportion of health practitioners who have received capacity-building support pertaining to climate change adaptation and health**	

*Definitional considerations*	This may be the most straightforward indicator, but it still requires specification. ‘Health practitioners’ needs explicit inclusion criteria: physicians, nurses, midwives, community health workers, allied health professionals, public health practitioners, or other categories. ‘Capacity-building support’ could include in-service training, pre-service curriculum, continuing professional development, or informal learning, with minimum duration or intensity thresholds to be specified. ‘Pertaining to climate change adaptation and health’ requires specification of what content qualifies: climate-health linkages generally, specific adaptation competencies, or emergency response training.
*Assessment approaches*	Depending on the definition of all concepts above, one indicator formula might be: (Health practitioners trained during a specified period/Total health practitioners practicing during the period) × 100. Key considerations include time frame (e.g., training in last two years as the expert group proposed, or ever trained), quality threshold (e.g., any exposure versus completion of accredited training programme), and competency assessment (e.g., inputs through training received versus outcomes through competency demonstrated).
*Data requirements and sources*	The denominator requires health workforce registries, training records professional licensing databases, or facility staffing records. The numerator requires training records, continuing professional development databases, or certification records. Many countries lack comprehensive training tracking systems, and self-reported data from surveys may be needed. Alternatively, a sampling strategy could be devised. At minimum, training programme attendance records or surveys-based estimates from representative samples could approximate coverage, through self-reported data and sampling limitations affect precision, and absence of competency assessment means training receipt does not equate to capability.
*Counterfactual and adaptation considerations*	The indicator should be complemented by outcome indicators to assess whether training translates to improved adaptive capacity and the degree to which capacity building for providers interacts with facility- and system-level preparedness.
*Implementation considerations*	This is relatively straightforward to implement compared to other indicators. However, it is a process indicator measuring training inputs rather than outcomes, training does not automatically translate to improved practice or health outcomes. Development of a core competency framework for climate-health adaptation, with tracking of training against this framework, would strengthen the indicator.

Before addressing each indicator individually, we establish a shared definitional framework for key terms. Readers are encouraged to consult these definitions alongside table 2, as many of the challenges identified in the indicator-specific analysis stem directly from terminological ambiguity in the adopted indicator text. The definitions provided are not prescriptive; rather, they illustrate the range of plausible interpretations that arise in the absence of standardised guidance, and the analytic consequences that follow from each.

An important aspect is the difference between association and attribution. **Association** refers to an observed statistical relationship between an exposure (e.g. extreme temperatures) and a health outcome (e.g. mortality). Association does not separate the underlying causes of those temperatures: it does not distinguish whether the heat stems from natural climate variability, anthropogenic climate change, or local urban effects.

**Attribution** is the ‘*process of evaluating the relative contributions of multiple causal factors to a change or event with an assessment of confidence’* [12]. Quantitative attribution employs a counterfactual approach linking the proportion of an association that can be attributed to a specific cause.

**Climate-related hazards** can be understood as climate-related (or weather) events or trends that ‘may cause loss of life, injury, or other health impacts, as well as damage and loss to property, infrastructure, livelihoods, service provision, ecosystems and environmental resources.’ [12], including heatwaves, droughts, hurricanes, and heavy precipitation. A **climate-sensitive hazard** might be slightly different and refers to hazards that are influenced, modulated, or exacerbated by climatic conditions, but where climate is not the sole or primary cause. The hazard exists independently of climate, yet its occurrence, intensity, geographical distribution, or timing is affected by climatic variables. It includes, for example, air pollution or ecosystems changes.

**Climate-sensitive** and **-related health outcomes** are those health outcomes (i.e. diseases, injuries, deaths) whose incidence or distribution might be affected by weather and climate variability, such as vector-borne diseases and heat-related morbidity and mortality. These outcomes can be tracked using combinations of exposure and health data.

**Climate change-related** and **-attributable health outcomes** represent the marginal or additional burden specifically attributable to anthropogenic climate change, capturing the long-term signal and requiring counterfactual analysis, comparing observed conditions to scenarios without anthropogenic climate change. These distinctions have profound implications for indicator design, data requirements, and interpretation of results.

**Counterfactual rates** refer to (i) observed rates associated with a historical baseline comparison scenario; (ii) modelled rates associated with a hypothetical baseline without climate change; or (iii) an exposure to a interventions that change the level of exposure to specific hazards.

**Adaptation actions** for health include a wide array of strategies and measures both within and outside the health sector.

**Vulnerability** can be understood as ‘The propensity or predisposition to be adversely affected. Vulnerability encompasses a variety of concepts and elements, including sensitivity or susceptibility to harm and lack of capacity to cope and adapt.’ [12]. Generally speaking, vulnerability has multiple dimensions including location and geographic exposure (e.g. populations in coastal, flood-prone, drought-affected, or urban heat island areas); socioeconomic vulnerability (e.g. low-income populations lacking adaptive capacity); physiological vulnerability (e.g. elderly, children, those with pre-existing mental health conditions); and occupational exposure (e.g. agricultural workers, outdoor workers, first responders). How to decompose and analyse vulnerability is a persistent challenge for the climate and health field.

**Resilience**, in this context, refers to the capacity of individuals, communities, institutions, or systems to anticipate, prepare for, respond to, and recover from climate-related hazards and stressors, whilst maintaining essential functions and adapting to long-term changes [13].

## 4. Pathways forward for the BAI for health

The multiple retrospective analyses synthesising scientific and technical guidance to political negotiations can inform the processes by which these indicators are operationalised and implemented. The Belém–Addis vision (2026–2028) provides a two-year policy alignment process and technical taskforce to improve metadata and methods. This represents an opportunity to address the challenges identified above. Success and long-term sustainability will require sustained engagement between technical experts and political processes, development of standardised methods acceptable across diverse national contexts, and investment in surveillance infrastructure, particularly in LMIC. Strategic investments in specific activities, such as development of essential adaptation interventions, surveillance of adaptation activities, and a standardised set of key counterfactual scenarios and disease burden estimates, will likely be required for successful translation and implementation.

The development of definitional guidance for key terms in each indicator is key, providing options that accommodate national contexts whilst enabling meaningful comparison. Guidance documents should specify minimum data requirements, analytical approaches, and quality assurance standards for each indicator. Pilot testing in diverse settings, including countries with strong surveillance systems and those with limited capacity, would identify practical implementation challenges and inform refinement.

In the long-term, strengthening vital registries and public health surveillance systems in countries with limited coverage is critical for mortality and morbidity indicators. Investment in health-climate data linkage infrastructure, including interoperability standards and data sharing agreements between health and meteorological sectors, addresses a critical implementation gap. Capacity building for epidemiological analysis of climate-health associations should be embedded in public health training programmes, and technical assistance mechanisms should support countries in implementing indicator tracking. Establishing independent monitoring mechanisms and civil society engagement, drawing on lessons from tobacco control and other domains where science faced policy resistance would strengthen accountability.

## 5. Conclusions

The analysis presented in this paper reflects one contribution to an emerging and necessarily collaborative dialogue in the climate-health sphere. As the Belém–Addis vision moves into its technical phase, the operationalisation of the BAI for health will require sustained engagement across scientific disciplines, public health practice, and policy processes. Meaningfully measuring adaptation progress demands that the science–policy interface function not as a series of successive handovers, but as an ongoing and iterative collaboration with careful consideration of how to effectively balance ambition with limited capacity. We present this perspective to call upon researchers, national health authorities, and negotiating Parties to actively engage in this process: the quality of global climate-health accountability will depend on the rigour of the choices made today.

## Data Availability

All data that support the findings of this study are included within the article (and any supplementary files). Supplementary data 1 available at https://doi.org/10.1088/1748-9326/ae6674/data1.
